# Supplementary material for: Molecular Modeling of Allosteric Site of Isoform-Specific Inhibition of the Peroxisome Proliferator-Activated Receptor PPARγ
Source: Biomolecules. 2022 Nov 1;12(11):1614. doi: 10.3390/biom12111614 (PMC9687262; doi:10.3390/biom12111614)
Supplement: Supplementary file 1 [file biomolecules-12-01614-s001.zip › biomolecules-1978181-supplementary.pdf]

Molecular Modeling of Allosteric Site of Isoform-Specific Inhibition of the Peroxisome Proliferator-activated Receptor PPARγ

Suliman Almahmoud[1*] and Haizhen A. Zhong[2*]

[1] Department of Medicinal Chemistry and Pharmacognosy, College of Pharmacy, Qassim University, Buraidah 51452, Saudi Arabia
[2] Department of Chemistry, University of Nebraska at Omaha, Omaha, Nebraska 68182, United States
* Correspondence: HAZ: hzhong@unomaha.edu; Tel.: (1) (402) 554-3145; co-Correspondence: SA; s.a.almahmoud@hotmail.com.

Table S1. The chemical structures of PPARγ antagonists.

Table S2. The chemical structures of PPARγ antagonists.

Figure S1. The chemical structures of PPARγ agonists.

Table S3. Glide docking scores (GScore, kcal/mol) of drug-like molecules extracted from a NCI database Against the PPARγ (2HFP) at allosteric site.

Table S4. Interacting residues of PPARγ antagonists at the orthosteric site.

Table S5. Interacting residues of PPARγ agonists at the orthosteric site and the allosteric site based on the Induced-Fit Docking.

Table S1. The chemical structures of PPARγ antagonists taken from Hopkins' paper [1].

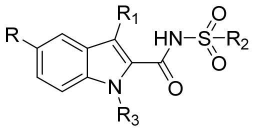

Compound	R	R_1	R_2	R_3
**NSI**	H	4-CH_3OPh	Ph	3-CF_3Bn
**1**	H	4-CH_3OPh	4-F-Ph	3-CF_3Bn
**2**	H	4-CH_3OPh	2-CO_2MePh	3-CF_3Bn
**3**	H	4-CH_3OPh	2-CH_3Ph	3-CF_3Bn
**4**	H	4-CH_3OPh	Me	3-CF_3Bn
**5**	H	H	Ph	3-CF_3Bn
**6**	H	H	4-F-Ph	3-CF_3Bn
**7**	H	H	4-Cl-Ph	3-CF_3Bn
**8**	H	H	3-CF_3-Ph	3-CF_3Bn
**9**	H	H	4-CH_3Ph	3-CF_3Bn
**10**	H	H	2-CH_3Ph	3-CF_3Bn
**11**	H	H	2-Naphthyl	3-CF_3Bn
**12**	H	H	2-CF_3-Ph	3-CF_3Bn
**13**	H	H	2-(5-Chlorothiophene)	3-CF_3Bn
**14**	H	H	Me	3-CF_3Bn
**15**	H	H	4-CF_3-Ph	4-CF_3Bn
**16**	H	H	3-CF_3-Ph	3-CH_3OBn
**17**	H	H	3-CF_3-Ph	3-CF_3OBn
**18**	H	H	3-CF_3-Ph	Et
**19**	H	H	3-CF_3-Ph	4-CF_3Bn
**20**	H	H	3-CF_3-Ph	3-BnOBn
**21**	H	H	3-CF_3-Ph	Bn
**22**	H	H	3-CF_3-Ph	2,5-DiClBn
**23**	H	H	3-CF_3-Ph	4-*t*-BuBn
**24**	Cl	H	3-CF_3-Ph	3-CF_3Bn
**25**	OBn	H	3-CF_3-Ph	3-CF_3Bn
**26**	OH	H	3-CF_3-Ph	3-CF_3Bn

Table S2. The chemical structures of PPARγ antagonists taken from Asteian's paper [2].

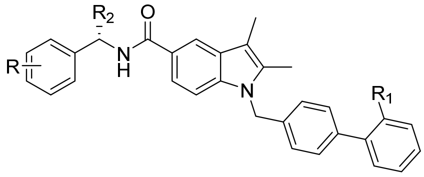

Compound	R	R_1	R2	Compound	R	R_1	R2
**27**	2-CH_3	COOH	H	**SR1664**	4-NO_2	COOH	CH_3
**28**	2-NH_2	COOH	H	**36**	3-iPr	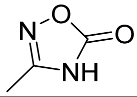	CH_3
**29**	2-NO_2	COOH	H	**37**	3-iPr	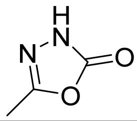	CH_3
**30**	3-iPr	COOH	CH_3	**38**	3-iPr	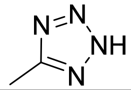	CH_3
**31**	3- 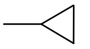	COOH	CH_3	**39**	3-iPr	CONHOH	CH_3
**32**	3-tBu	COOH	CH_3	**40**	3-iPr	$CONH_2$	CH_3
**33**	4-iPr	COOH	CH_3	**41**	3-iPr	$CON(CH_3)_2$	CH_3
**34**	4-tBu	COOH	CH_3	**42**	3-iPr	COOH	CF_3
**35**	3- 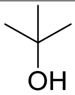	COOH	CH_3	**43**	3-iPr	COOH	3-iPr

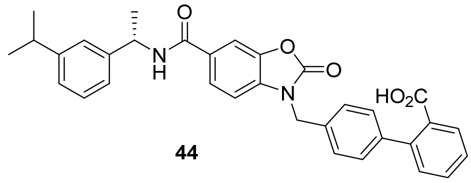

44

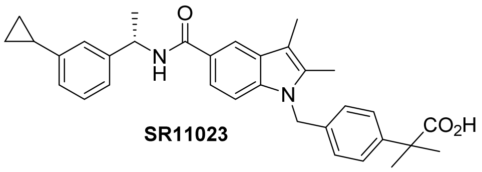

SR11023

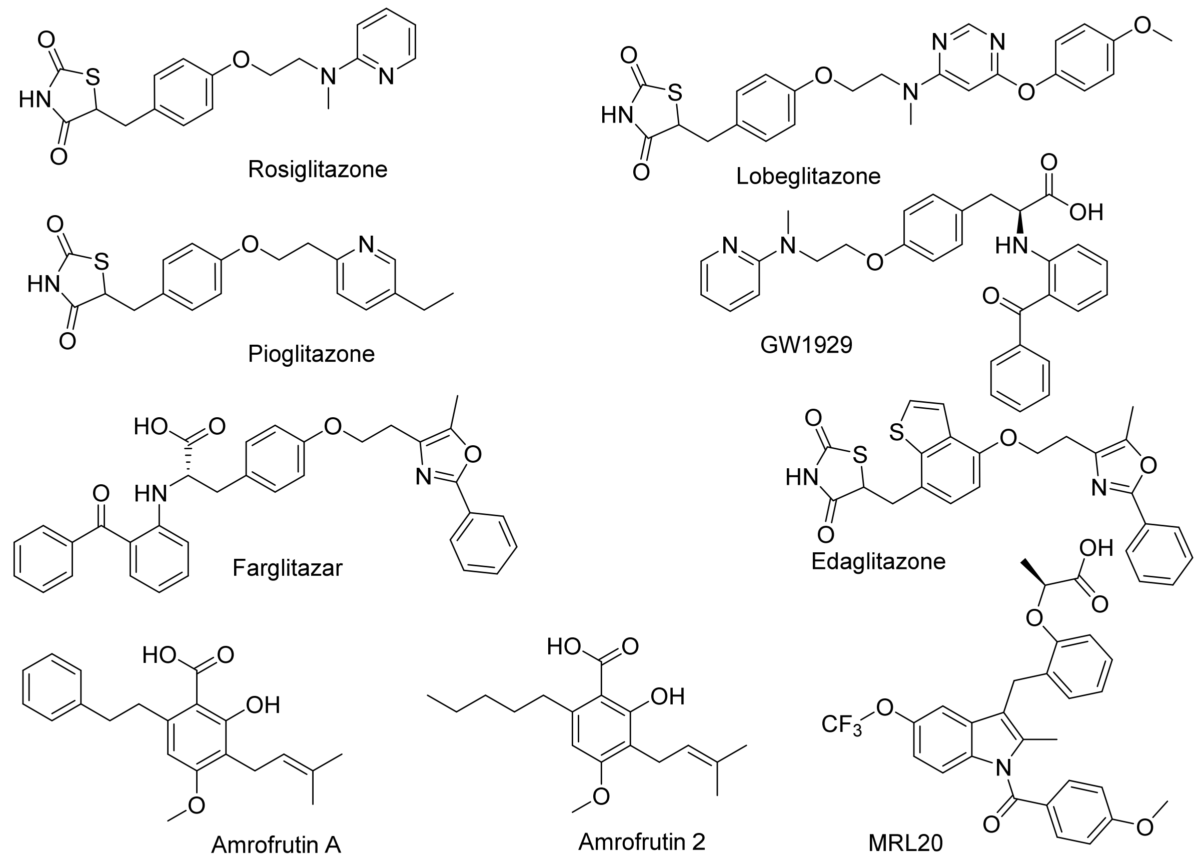

Figure S1. The chemical structures of PPARγ agonists.

Table S3. Glide docking scores (GScore, kcal/mol) of drug-like molecules extracted from a NCI database Against the PPARγ (2HFP) at allosteric site.

NCI ID	GScore	NCI ID	GScore	NCI ID	GScore	NCI ID	GScore	NCI ID	GScore
**116**	-6.57	**47742**	-8.92	**95895**	-5.44	**130813**	-8.28	**171538**	-6.30
**153**	-6.13	**48151**	-7.65	**99547**	-8.54	**130842**	-5.41	**172540**	-7.21
**3098**	-3.65	**49628**	-6.92	**101523**	-8.59	**131366**	-8.68	**172846**	-7.50
**6918**	-5.84	**51478**	-5.27	**101672**	-6.10	**131367**	-8.92	**177363**	-7.99
**7579**	-7.68	**52003**	-6.80	**101793**	-8.08	**132263**	-7.31	**178023**	-7.99
**8008**	-7.78	**53199**	-5.51	**103843**	-7.19	**132898**	-7.22	**178889**	-6.78
**8490**	-5.87	**54906**	-6.76	**105274**	-2.80	**134118**	-6.13	**180617**	-7.27
**8609**	-8.76	**56243**	-8.84	**105337**	-6.56	**135634**	-6.89	**180627**	-7.71
**8611**	-9.12	**57034**	-10.75	**106156**	-9.66	**135848**	-9.42	**180642**	-6.90
**9011**	-4.64	**60616**	-7.64	**106221**	-6.73	**139461**	-7.25	**180648**	-7.63
**9349**	-7.28	**62571**	-5.89	**110347**	-8.63	**141337**	-5.53	**180661**	-7.17
**10406**	-6.33	**63837**	-7.31	**110559**	-3.91	**142333**	-7.60	**190528**	-6.80
**10671**	-9.92	**66072**	-4.71	**111593**	-5.81	**143351**	-7.88	**190748**	-6.50
**12985**	-7.19	**69584**	-9.17	**112828**	-7.57	**143748**	-8.31	**201618**	-7.27
**13481**	-8.36	**70824**	-6.88	**113311**	-6.99	**144441**	-6.86	**201666**	-7.58
**13843**	-8.52	**70914**	-10.38	**113915**	-6.69	**146005**	-5.81	**202677**	-7.88
**14013**	-9.08	**70972**	-6.44	**114133**	-6.67	**147980**	-6.70	**204583**	-6.64
**15285**	-8.80	**71012**	-5.49	**114631**	-8.18	**148001**	-6.77	**204590**	-6.52
**15903**	-6.04	**71682**	-7.25	**114673**	-6.33	**149543**	-5.98	**205546**	-7.69
**16764**	-8.15	**71790**	-6.86	**114819**	-7.01	**150206**	-5.53	**205684**	-8.44
**16765**	-8.15	**72559**	-8.90	**117195**	-6.40	**150386**	-8.82	**205811**	-9.58
**16768**	-9.63	**74975**	-5.98	**117777**	-4.40	**151215**	-7.83	**210344**	-9.86
**19223**	-6.33	**77700**	-4.04	**119707**	-7.51	**152426**	-8.00	**211226**	-8.04
**21312**	-7.07	**77720**	-6.21	**119765**	-6.67	**155243**	-8.07	**211831**	-7.32
**24914**	-6.17	**79023**	-9.82	**120183**	-8.89	**156601**	-9.99	**212018**	-8.10
**26870**	-8.66	**79566**	-5.62	**122309**	-6.98	**156773**	-7.17	**212032**	-9.16
**27265**	-7.41	**81129**	-5.22	**122514**	-6.38	**157402**	-4.63	**212112**	-5.07
**30019**	-8.80	**84538**	-7.53	**123112**	-7.94	**157967**	-6.14	**213899**	-7.67
**30967**	-8.94	**85154**	-6.82	**123222**	-6.57	**161387**	-7.11	**215556**	-9.97
**31546**	-6.82	**85267**	-7.91	**123247**	-5.06	**162183**	-6.11	**216451**	-7.77
**33408**	-6.04	**85410**	-6.26	**123976**	-8.04	**162501**	-9.45	**216506**	-6.50
**34567**	-7.56	**85518**	-7.29	**124442**	-8.51	**162765**	-6.29	**216694**	-7.70
**36487**	-6.69	**85679**	-7.80	**125281**	-7.81	**163369**	-4.53	**217080**	-7.22
**36506**	-10.26	**85740**	-5.44	**125661**	-5.16	**164089**	-10.94	**226116**	-7.55
**37117**	-7.73	**86711**	-8.34	**126424**	-5.30	**164113**	-5.63	**226178**	-8.16
**37815**	-8.77	**88972**	-5.92	**127678**	-8.73	**164377**	-7.48	**226517**	-9.62
**39006**	-6.08	**89808**	-8.49	**127690**	-6.39	**164929**	-6.86	**227395**	-4.90
**39612**	-8.07	**91332**	-10.58	**128094**	-4.94	**165995**	-5.63	**230297**	-11.56
**42399**	-3.86	**91397**	-8.77	**128583**	-6.90	**167739**	-6.07	**231315**	-9.15
**44491**	-8.35	**92204**	-7.31	**128594**	-9.96	**167819**	-7.97	**231923**	-6.49
**45093**	-5.71	**92228**	-6.56	**130101**	-8.92	**170757**	-7.31	**232022**	-7.76
**234698**	-9.18	**341661**	-10.03	**515421**	-6.74	**630981**	-4.02	**667386**	-6.67
**240360**	-7.78	**343544**	-8.27	**523235**	-6.63	**632016**	-7.68	**667708**	-8.21
**244974**	-8.87	**344240**	-9.77	**527570**	-5.76	**632242**	-7.23	**671438**	-7.18
**254681**	-9.24	**345725**	-6.28	**529321**	-8.55	**633057**	-8.81	**672084**	-9.87
**269193**	-5.06	**346878**	-7.48	**602681**	-7.58	**633971**	-6.54	**672293**	-6.93
**270150**	-7.33	**349963**	-8.74	**603656**	-6.02	**637660**	-5.35	**674002**	-6.56
**270734**	-7.73	**350111**	-7.52	**608550**	-6.07	**638038**	-8.44	**674012**	-7.49
**273905**	-9.93	**350986**	-6.01	**609357**	-6.18	**639829**	-7.75	**674612**	-6.47

**277480**	-6.71	**353485**	-7.82	**609523**	-7.09	**640341**	-8.46	**675766**	-7.19
**279829**	-8.74	**356465**	-8.40	**610540**	-6.14	**640991**	-7.84	**675767**	-7.98
**281299**	-6.16	**356785**	-6.84	**612475**	-11.78	**640996**	-8.47	**676464**	-6.49
**284669**	-6.31	**357681**	-7.99	**612977**	-6.44	**641217**	-7.11	**676607**	-10.51
**289626**	-8.76	**359827**	-7.77	**613586**	-9.79	**641250**	-9.53	**677157**	-8.56
**291629**	-7.83	**363784**	-8.54	**613748**	-9.15	**641602**	-8.05	**679497**	-8.87
**292943**	-8.22	**363918**	-7.99	**617013**	-7.05	**642075**	-9.13	**679513**	-7.98
**294866**	-6.30	**364069**	-5.18	**618178**	-9.15	**642305**	-9.36	**680329**	-6.28
**296242**	-7.28	**364385**	-6.65	**618443**	-8.03	**642324**	-9.54	**681170**	-7.55
**298141**	-6.91	**365357**	-9.29	**618449**	-7.27	**643509**	-8.32	**681536**	-6.23
**299208**	-6.69	**366098**	-10.03	**618555**	-7.66	**644965**	-8.06	**681633**	-9.27
**299236**	-6.60	**367933**	-9.70	**618660**	-6.12	**645168**	-7.72	**681958**	-8.38
**300910**	-5.19	**368279**	-9.58	**618682**	-7.14	**645980**	-7.98	**682500**	-5.65
**304891**	-8.56	**371012**	-7.01	**618688**	-7.39	**646474**	-8.47	**682506**	-9.00
**308001**	-12.89	**371194**	-9.22	**619196**	-8.95	**647592**	-7.47	**683237**	-6.63
**309842**	-6.26	**371488**	-6.25	**620478**	-5.46	**648635**	-7.81	**683711**	-5.60
**310324**	-7.03	**372059**	-6.86	**622586**	-6.06	**648639**	-6.49	**685509**	-8.20
**310361**	-6.11	**372302**	-8.31	**622958**	-7.87	**648650**	-8.51	**685706**	-7.53
**310835**	-6.76	**372523**	-6.23	**623091**	-7.44	**648689**	-4.84	**687105**	-5.60
**313957**	-7.85	**372642**	-7.95	**623712**	-6.35	**651331**	-2.48	**687524**	-7.88
**317877**	-6.57	**374682**	-6.69	**623768**	-8.57	**652035**	-6.80	**687739**	-7.86
**318822**	-6.85	**375722**	-8.47	**624333**	-6.88	**652861**	-8.23	**687869**	-8.80
**319112**	-6.44	**379100**	-11.92	**624425**	-8.16	**652900**	-7.40	**688818**	-9.21
**319688**	-6.66	**379472**	-6.59	**624454**	-8.06	**654387**	-5.83	**690564**	-8.85
**320204**	-6.91	**380509**	-6.54	**624546**	-7.25	**655041**	-5.69	**691348**	-7.57
**320212**	-7.15	**381584**	-7.28	**624547**	-7.74	**656455**	-7.52	**691350**	-7.51
**320562**	-7.93	**400063**	-6.50	**624548**	-6.76	**657990**	-7.19	**691424**	-8.98
**320864**	-7.16	**401688**	-7.98	**627785**	-5.81	**658262**	-11.55	**691578**	-6.86
**325304**	-10.66	**401696**	-9.32	**627889**	-7.93	**658994**	-9.12	**693055**	-5.53
**328102**	-7.03	**401828**	-6.81	**629819**	-11.91	**659343**	-8.01	**693123**	-6.76
**331921**	-7.88	**403031**	-4.68	**629827**	-6.15	**661081**	-7.54	**694919**	-6.36
**333346**	-5.14	**405904**	-8.03	**630312**	-6.25	**662564**	-5.93	**695857**	-6.62
**335415**	-7.73	**406369**	-6.67	**630321**	-7.18	**664889**	-6.47	**731365**	-7.04
**335994**	-10.45	**408562**	-9.79	**630373**	-9.79	**665312**	-7.94	**338620**	-7.10
**337738**	-8.06	**409253**	-6.93	**512601**	-5.48	**666613**	-7.16		
**338540**	-8.51	**409615**	-7.79	**514213**	-6.78	**666714**	-7.03		

Table S4. Interacting residues of PPARγ antagonists at the orthosteric site.

Compound	Interacting Residues	Compound	Interacting Residues
**NSI**	Phe282, Phe363, Lys367, His449	**22**	Phe282, Phe363, Lys367
**SR1664**	Lys265, Ser289, Ser342, Lys367	**23**	Phe363
**SR11023**	Lys265, Ser289, Ser342	**24**	Phe282, Lys367, His449
**1**	Phe282, Phe363, Lys367, His449	**25**	Phe363
**2**	Phe282, His449	**26**	Phe282, Lys367
**3**	Phe282, Phe363, His449	**27**	Lys265, Ser289, Ser342
**4**	Lys367, His449	**28**	Lys265, Ser342
**5**	Lys367	**29**	Lys265, Ser289, Ser342
**6**	His449	**30**	Lys265, Ser289, Ser342
**7**	Phe282, Lys367, His449	**31**	Lys265, Phe282, Ser342
**8**	Phe282, Lys367, His449	**32**	Lys265, Phe282, Ser342
**9**	Phe282, His449	**33**	Lys265
**10**	Phe363, Lys367, His449	**34**	Lys265, Phe282, Ser289, Ser342
**11**	Phe282, Lys367	**35**	Lys265, Ser342, Tyr473
**12**	Lys367	**36**	Lys265, Ser342
**13**	Lys367, His449	**37**	Lys265, Phe282
**14**	Phe282, Lys367, His449	**38**	Lys265
**15**	Phe282, Lys367, His449	**39**	Lys265, Ser342
**16**	Phe282, Phe363, Lys367	**40**	Lys265, Glu291
**17**	Phe282, Lys367	**41**	Lys265, Gly284, Tyr327
**18**	Phe282, Lys367	**42**	Lys265
**19**	Phe282, Lys367	**43**	Lys265
**20**	NA	**44**	NA
**21**	Phe282, Phe363, Lys367		

Table S5. Interacting residues of PPARγ agonists at the orthosteric site and the allosteric site based on the Induced-Fit Docking.

Title	Ortho	Allo
**Rosiglitazone**	Ser289, His323, Gln286, Lys367	His323, Tyr327, Tyr473
**Lobeglitazone**	Ser289, His323, Gln286, Lys367	Lys265, Tyr473, Tyr327
**Pioglitazone**	Ser289, His323, Gln286, Lys367	His323, Tyr327, Tyr473, Leu340
**GW1929**	Leu340, Glu343, Lys265	HIE266, LYS265, Ser342
**Farglitazar**	Ser342	HIE266, LYS265, Ser342
**Edaglitazone**	Ser342	His323, Tyr327, Tyr473
**Amorfrutin 1**	Ser342, Glu343, Arg288	Glu343, Lys265
**Amorfrutin 2**	Ser342, Glu343, Arg288	Ser342
**MRL20**	Ser342	Ser342

References

1. Hopkins, C.R.; O'Neil, S.V.; Laufersweiler, M.C.; Wang, Y.; Pokross, M.; Mekel, M.; Evdokimov, A.; Walter, R.; Kontoyianni, M.; Petrey, M.E.; et al. Design and synthesis of novel N-sulfonyl-2-indole carboxamides as potent PPAR-γ binding agents with potential application to the treatment of osteoporosis. *Bioorganic Med. Chem. Lett.* **2006**, *16*, 5659–5663. https://doi.org/10.1016/j.bmcl.2006.08.003.
2. Asteian, A.; Blayo, A.-L.; He, Y.; Koenig, M.; Shin, Y.; Kuruvilla, D.S.; Corzo, C.A.; Cameron, M.D.; Lin, L.; Ruiz, C.; et al. Design, Synthesis, and Biological Evaluation of Indole Biphenylcarboxylic Acids as PPARγ Antagonists. *ACS Med. Chem. Lett.* **2015**, *6*, 998–1003. https://doi.org/10.1021/acsmedchemlett.5b00218.
